# Mazabraud Syndrome: The Contribution of Interventional Physiatry in a Clinical Case With 10 Years of Evolution

**DOI:** 10.7759/cureus.101376

**Published:** 2026-01-12

**Authors:** Alexandra Lagarto, Sandra Assunção, Sara Ribeiro Silva, Rodrigo Correia, José Luís Carvalho, Daniela Costa Martins

**Affiliations:** 1 Physical Medicine and Rehabilitation, Unidade Local de Saúde da Região de Leiria, Leiria, PRT; 2 Neuromuscular Surgery and Rehabilitation, Centro de Medicina de Reabilitação da Região Centro – Rovisco Pais, Unidade Local de Saúde de Coimbra, Tocha, PRT; 3 Physical Medicine and Rehabilitation, Unidade Local de Saúde de Entre o Douro e Vouga, Santa Maria da Feira, PRT; 4 Intervention and Musculoskeletal Rehabilitation Unit, Centro de Reabilitação do Norte, Unidade Local de Saúde Gaia e Espinho, Vila Nova de Gaia, PRT

**Keywords:** case report, fibrous dysplasia, intramuscular myxoma, mazabraud syndrome, minimally invasive procedure, radiofrequency ablation, sacroiliac joint pain

## Abstract

Mazabraud syndrome is a rare condition characterized by fibrous dysplasia and intramuscular myxomas. We present the case of a 66-year-old woman with chronic lumbosacral pain and imaging findings consistent with this syndrome, confirmed by biopsy. After years of conservative management with stable lesions but persistent symptoms, she underwent fluoroscopy-guided radiofrequency neurotomy of sacroiliac innervation, achieving complete pain relief and functional recovery. The patient was followed for five months after the intervention, with sustained symptom control and functional improvement. This case illustrates the diagnostic challenge and underscores the novelty of a targeted, minimally invasive interventional approach for symptom control in Mazabraud syndrome.

## Introduction

Mazabraud syndrome is a rare form of fibrous dysplasia associated with intramuscular myxomas, with approximately 100 cases reported in the literature [1-3]. Two-thirds of cases occur in women, and fibrous dysplasia typically presents around the age of 37 years, whereas myxomas typically present at 47 years [1,2,4].

Fibrous dysplasia is characterized by the replacement of normal bone and bone marrow with abnormal fibrous tissue [2,5]. It may be asymptomatic or present with deformities, pain, pathological fractures, or functional impairment [1,4,5]. It most frequently affects the pelvis and fémur [2], and can be monostotic or polyostotic, with the polyostotic form being more prevalent [4]. Intramuscular myxomas are rare benign neoplasms characterized by the proliferation of myxoid connective tissue. They may be asymptomatic or present as painless muscular masses [1]. These lesions are usually located in the lower limbs, commonly involving the quadriceps muscle, hip adductors, and gluteus muscles, and may occur as single or multiple lesions [4]. Bone lesions often precede the appearance of myxomas, and the coexistence of both entities (fibrous dysplasia and myxomas) should always raise suspicion for Mazabraud syndrome, even when the lesions appear at different times [1,3]. Given the frequent involvement of the pelvis and proximal femur, Mazabraud syndrome may contribute to chronic lumbosacral or sacroiliac joint pain, which can significantly impact function and quality of life.

## Case presentation

A 66-year-old woman with a history of arterial hypertension, type 2 diabetes mellitus, non-alcoholic fatty liver disease, dyslipidemia, obesity (body mass index: 32 kg/m²), and anxiety-depressive disorder was referred to the Physical and Rehabilitation Medicine (PRM) Interventional consultation, dedicated to musculoskeletal diagnosis and minimally invasive procedures. The reason for referral was right-sided lumbosacral pain with a 10-year history for clinical assessment and consideration of pain relief treatment.

The symptoms began in 2015, with referred pain to the ipsilateral gluteal region and the superolateral aspect of the thigh, without any history of trauma, predominantly mechanical, with occasional exacerbations reaching a maximum of 10/10 on the Numeric Pain Rating Scale (NPRS), partially relieved by ibuprofen 600 mg twice daily. Functionally, regarding basic activities of daily living (BADL), the patient had difficulty caring for the lower limb extremities, and in terms of instrumental activities of daily living (IADL), she was unable to perform household cleaning.

On physical examination, sacroiliac compression and distraction tests, FABER-Patrick, and Gaenslen tests were positive with pain referred to the right sacroiliac joint. There was mild tenderness over the greater trochanter and along the gluteal muscles, without muscle strength or sensory deficits in the lower limbs. Radiculopathy was excluded as specific maneuvers for nerve root irritation were negative, and hip joint pathology was ruled out, given preserved range of motion without pain during targeted tests.

**Figure 1:**
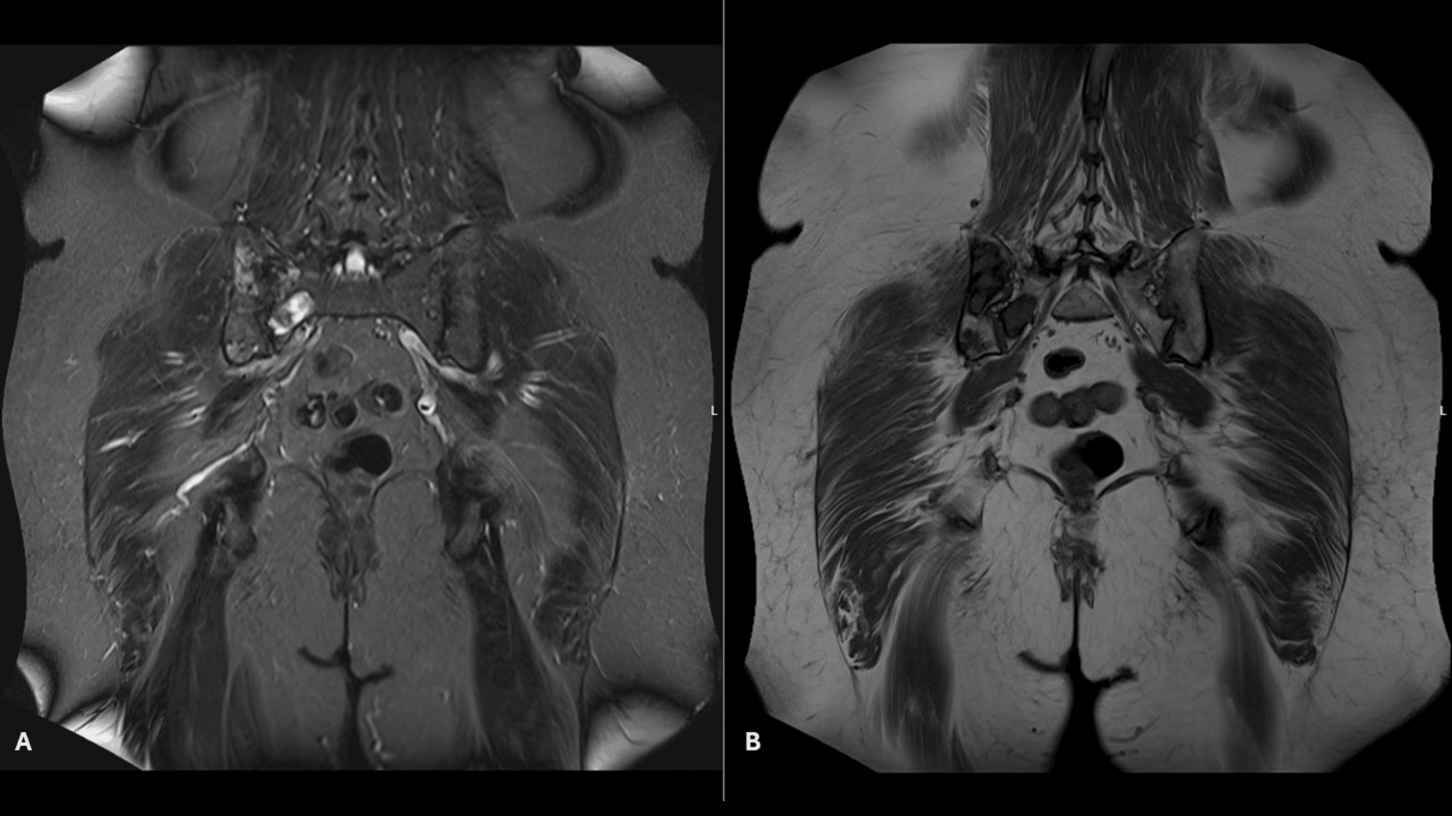
MRI of fibrous dysplasia lesions. Fibrous dysplasia lesions in the right sacral ala and adjacent right ilium. Coronal T2-weighted image on the left (A) and T1-weighted image on the right (B) showing hyperintense and hypointense signals, respectively.

MRI revealed two bone lesions in the right sacral ala and adjacent portion of the right ilium, hypointense with a ground-glass appearance on T1-weighted sequences and hyperintense on T2-weighted sequences, well circumscribed by a hypointense rim, without aggressive features, with well-defined borders, suggestive of fibrous dysplasia (Figure 1). In the surrounding area, within the right gluteus minimus and maximus muscles, two well-circumscribed oval formations were identified, hyperintense on T2 and short tau inversion recovery, hypointense on T1, and showing contrast enhancement, consistent with myxoma. Given the coexistence of bone lesions compatible with fibrous dysplasia and soft tissue myxomas, Mazabraud syndrome was suspected. A biopsy of the right gluteal region confirmed the nature of the lesion: a low-grade myxoid neoplasm, paucicellular, with thin-walled vessels and no cytological atypia.

The patient remained under clinical and imaging follow-up for 10 years, with imaging stability and persistence of pain and functional limitations. During this period, structured conservative rehabilitation was attempted, including physical therapy focused on core stabilization and pelvic alignment, combined with pharmacological management (nonsteroidal anti-inflammatory drugs). These measures provided only partial and temporary relief, with persistent functional impairment, which justified consideration of interventional treatment.

At the first PRM consultation, after obtaining informed consent, an ultrasound-guided anesthetic block of the right L5 dorsal ramus and the right S1, S2, and S3 lateral branches was performed; the patient reported immediate complete pain relief at the time.

**Figure 2:**
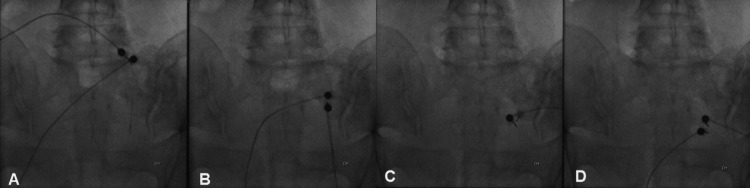
Fluoroscopic guidance during thermal radiofrequency procedure. (A) Bipolar radiofrequency ablation of the right L5 dorsal ramus. (B and D) Bipolar radiofrequency ablation of the right S1 and S3 lateral branches. (C) Unipolar radiofrequency ablation of the right S2 lateral branch.

Subsequently, she underwent bipolar thermal radiofrequency (RF) ablation of the right L5 dorsal ramus and the right S1 and S3 lateral branches; at S2, only unipolar thermal RF was possible due to technical difficulties. With the patient in the prone position, two 22-gauge electrodes, 100 mm in length, 10 mm active tip, were inserted immediately lateral to the right L5-S1 facet joint, arching over the ala of the sacrum, and at the level of each right S1, S2, and S3 foramen, under uniplanar fluoroscopic guidance (Figure 2). Thermal RF neurotomy was performed at 80°C for 90 seconds, with local anaesthetic (0.5 mL of 2% lidocaine per level) injected immediately before ablation and 1 mL of 0.2% ropivacaine after the procedure. Following the intervention, the patient was monitored in the recovery area for approximately 30 minutes and discharged after reassessment, presenting with complete pain resolution. No adverse events were identified.

At follow-up, seven weeks after the procedure, the patient was asymptomatic. She reported no pain, except for occasional mild right sacroiliac discomfort (1/10 on NPRS) during activities such as lifting heavy objects. She discontinued oral analgesia (ibuprofen 600 mg twice daily) since the procedure and had no limitations in BADL or IADL. On examination, sacroiliac compression, sacroiliac distraction, and Gaenslen tests were negative; FABER-Patrick elicited mild discomfort in the right hip joint; there was no tenderness over the greater trochanter or gluteal region; and she had no motor or sensory deficits in the lower limbs. The Oswestry Disability Index (ODI) [6] score was 41% before RF, indicating severe disability, and 0% after the procedure, indicating minimal disability.

A follow-up telephone evaluation was performed five months after the procedure, and the patient remained asymptomatic, without the need for analgesics, with no limitations in daily activities, and with an ODI score of 0%.

**Figure 3:**
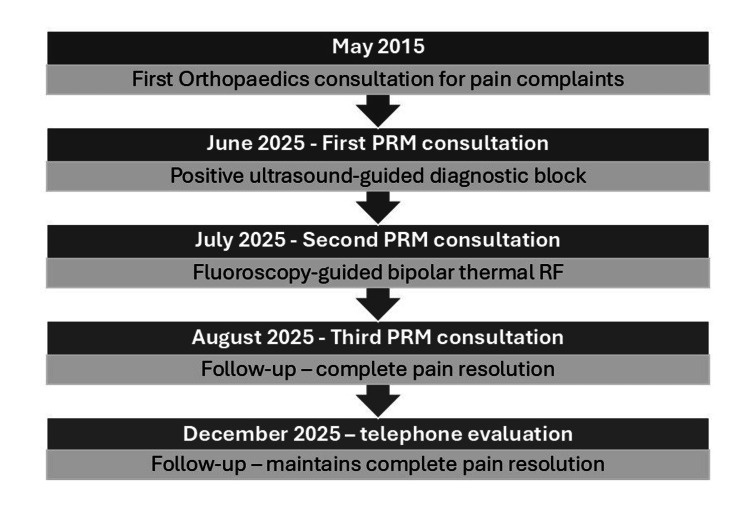
Timeline of all medical evaluations and procedures. PRM = Physical and Rehabilitation Medicine; RF = radiofrequency

A timeline of all medical evaluations and procedures is shown in Figure 3.

## Discussion

Mazabraud syndrome is a rare clinical entity characterized by the association of fibrous dysplasia and intramuscular myxomas [1,3]. Diagnosis is primarily clinical and imaging-based; however, its variable presentation can pose diagnostic challenges, and biopsy may be useful to exclude malignancy [1,3].

Imaging plays a key role, with MRI being the modality of choice for diagnosing Mazabraud syndrome, as it demonstrates the coexistence of both bone and soft tissue lesions. Bone lesions typically appear hypointense on T1-weighted sequences and hyperintense on T2, often with a peripheral low-signal rim on all sequences [1]. Myxomas present as well-defined masses, isointense to muscle on T1 and hyperintense on T2, usually showing intense and heterogeneous contrast enhancement [7]. In this patient, the presence of two well-defined bone lesions associated with intramuscular lesions in the same anatomical region strongly suggested this diagnosis. Biopsy ruled out malignancy and confirmed the diagnosis.

Treatment is generally conservative, with regular follow-up due to the low but existing risk of malignant transformation of bone lesions [3,8]. Surgery may be indicated in cases of persistent pain, fracture, or significant deformity [3]. Bisphosphonate use has been reported for symptom control and lesion stabilization, although evidence remains limited and based on case reports [1,9]. In this patient, conservative management with imaging surveillance was appropriate, given the benign behavior of the lesions.

Conventional RF neurotomy for denervation has emerged as an effective minimally invasive approach for managing chronic sacroiliac pain [10]. This technique applies electromagnetic waves to generate an electric current, producing heat. In chronic pain, RF currents delivered through an electrode placed near a nociceptive sensory nerve create a thermal lesion, thereby interrupting pain transmission [11]. Careful patient selection is essential, with RF denervation of the sacroiliac joint indicated only after a positive response to diagnostic anesthetic blocks [12]. This procedure has a favorable safety profile [13] and can be repeated if symptoms recur [12].

In this patient, fluoroscopy-guided conventional thermal RF targeting sensory branches for pain control, following a positive diagnostic block, resulted in complete symptom resolution, eliminating functional limitations and improving quality of life, as confirmed by the ODI [6] score, while avoiding more invasive surgical procedures, particularly relevant in patients with multiple comorbidities. To our knowledge, this is the first reported case of Mazabraud syndrome treated with RF neurotomy for sacroiliac pain, highlighting the innovative use of a well-established technique in a rare musculoskeletal condition.

This report is limited by its single-case design, which precludes definitive conclusions regarding efficacy and reproducibility. Outcomes may vary. Despite this, the successful outcome suggests that targeted RF neurotomy could be considered for symptom control in other rare musculoskeletal or bone-related pain conditions where conventional management fails, provided that careful diagnostic confirmation and patient selection are ensured.

This case highlights the long-term follow-up of Mazabraud syndrome with imaging stability and absence of complications, histological confirmation, and a favorable response to a novel, minimally invasive technique for this condition.

## Conclusions

In a rare syndrome marked by the coexistence of fibrous dysplasia and intramuscular myxomas, the management of chronic pain can be challenging. This case illustrates a practical, stepwise approach: clinical suspicion supported by MRI, histological confirmation when indicated, conservative surveillance for lesion stability, and targeted intervention when pain persists. Fluoroscopy‑guided RF denervation of sacroiliac sensory branches, performed after a positive diagnostic block, proved safe, well-tolerated, and clinically effective, restoring function and obviating the need for surgical procedures. These findings reinforce the importance of careful patient selection and rigorous procedural technique in achieving meaningful outcomes and support RF neurotomy as a viable option for refractory sacroiliac pain in Mazabraud syndrome. Further case reports or case series are encouraged to validate these findings and explore their applicability to similar clinical contexts.
